# The efficiency of a customized distalizer with Variety SP^®^ screws anchored on palatal miniscrews for upper molar distalization

**DOI:** 10.1590/2177-6709.29.2.e2423253.oar

**Published:** 2024-06-10

**Authors:** Davit POGHOSYAN, Davit GRIGORYAN, Diana TER-POGHOSYAN, Gokulraj GUNAVEERASEKARAN, Swasa DARA, Hrant TER-POGHOSYAN

**Affiliations:** 1 Yerevan State Medical University, Department of Pediatric Dentistry and Orthodontics (Yerevan, Armenia).

**Keywords:** Cephalometrics, Class II, Distalization, Orthodontic miniscrew, Cefalometria, Classe II, Distalização, Mini-implante ortodôntico

## Abstract

**Objective:**

To assess the effectiveness of a customized distalizer with Variety SP^®^ screws anchored on palatal miniscrews for upper molar distalization.

**Methods:**

Seventeen patients aged between 12.5 and 24 years underwent distalization with a customized distalizer. Lateral cephalogram and cast analysis were performed before and after distalization. Linear and angular parameters of the upper first molar, first premolar, and central incisor were assessed.

**Results:**

Distalization with the force passing near the center of resistance (CR_es_) of the upper first molars resulted in distal movement, with minimal distal tipping (2.8* *±* *0.45°, *p*<* *0.05). However, distalization passing occlusal to the CR_es_ led to greater distal tipping (13.6* *±* *1.63°, *p*<* *0.05). Statistically significant spontaneous distal tipping and distal movement of the upper first premolars occurred, with a mean of 6.2* *±* *1.24° (*p*<* *0.05) and 0.68* *±* *0.34 mm (*p*<* *0.05), respectively. The positional change of the upper central incisors presented a mean of -0.23* *±* *0.1 mm (*p*>* *0.05) and 2.65* *±* *1.1° (*p*<* *0.05). Upper first molar intrusion was statistically significant, with a mean of 0.88* *±* *0.2 mm (*p*<* *0.05).

Upper right and left first molars rotation towards palatal midline presented mean of 4.1* *±* *0.19° (*p*<* *0.05) and 3.4 * *±* *0.1° (*p*<* *0.05), respectively. Additionally, the distance between upper right and left first molars increased significantly, with a mean of 2.54* *±* *0.01 mm (*p*<* *0.05).

**Conclusion:**

The study successfully demonstrated the efficiency of molar distalization without anchorage loss using a customized distalizer anchored on palatal miniscrews.

## RESUMO

**Objetivo:**

Avaliar a eficácia de um distalizador personalizado com parafusos Variety SP^®^ ancorado em mini-implantes palatinos para distalização de molares superiores.

**Métodos:**

Dezessete pacientes com idade entre 12,5 e 24 anos foram submetidos à distalização com o distalizador customizado. Análises das radiografias cefalométricas laterais e dos modelos de gesso foram realizadas antes e depois da distalização. Foram avaliados parâmetros lineares e angulares dos primeiros molares, primeiros pré-molares e incisivos centrais superiores.

**Resultados:**

A distalização com a força passando perto do centro de resistência (CRes) dos primeiros molares superiores resultou em distalização com inclinação mínima (2,8 ± 0,45°, *p*<* *0,05). Entretanto, a distalização passando oclusal ao CRes levou a uma maior inclinação para distal (13,6 ± 1,63°, *p*<* *0,05). Ocorreu inclinação distal espontânea e movimento distal estatisticamente significativo dos primeiros pré-molares superiores, com média de 6,2 ± 1,24° (*p*<* *0,05) e 0,68 ± 0,34 mm (*p*<* *0,05), respectivamente. A mudança de posicionamento dos incisivos centrais superiores apresentou média de -0,23 ± 0,1 mm (*p*>* *0,05) e 2,65 ± 1,1° (*p*<* *0,05). A intrusão dos primeiros molares superiores foi estatisticamente significativa, com média de 0,88 ± 0,2 mm (*p*<* *0,05). A rotação dos primeiros molares superiores direito e esquerdo em direção à linha média palatina apresentou média de 4,1 ± 0,19° (*p*<* *0,05) e 3,4 ± 0,1° (*p*<* *0,05), respectivamente. Além disso, a distância entre os primeiros molares superiores direito e esquerdo aumentou significativamente, com média de 2,54 ± 0,01 mm (*p*<* *0,05).

**Conclusão:**

O estudo demonstrou com sucesso a eficiência da distalização de molares sem perda de ancoragem utilizando um distalizador customizado ancorado em mini-implantes palatinos.

## INTRODUCTION

Distalization of upper molars as a method for correction of upper crowding is getting more and more popular as an alternative to premolar extraction. Despite the large number of molar distalizing devices, orthodontists prefer compliance-free intraoral bonded appliances. There are many types of intraoral distalizers known by orthodontists, for example: Distal Jet, Pendulum, Jones Jig, iPanda, etc. Many of them have disadvantages, such as loss of anchorage, molar tipping and rotation during distalization and compromised oral hygiene. The Pendulum appliance, introduced by Dr. James Hilgers in 1992, is one of the most popular options. Various modifications of the Pendulum appliance exist today. Byloff et al.^1^ conducted a study to evaluate the dental and skeletal effects of the Pendulum appliance. The results demonstrated that the pendulum appliance moved molars distally without creating a dental or skeletal bite opening, with little anchorage loss. However, a significant amount of molar tipping should be considered when using this appliance. In the study of Bussick et al.^2^, the mean distalization with the Pendulum appliance was 5.7 mm.

Intraoral distalization appliances typically comprise a distalizing mechanism and anchor teeth. To prevent anchorage loss during distalization, orthodontists have started using distalization appliances in combination with palatal miniscrews. A study by Kinzinger et al.^3^ evaluated the effectiveness of a skeletonized distal jet appliance for maxillary molar distalization. In the area of the cement-enamel junction, the permanent first molars were distalized by a mean of 3.92 ± 0.53 mm and intruded by a mean of 0.16 ± 0.26 mm. At the same time, they experienced distal tipping of 2.79 ± 2.51°. The first premolars, included in the anchorage setup, mesialized by 0.72 ± 0.78 mm and simultaneously tipped by 1.15 ± 2.98° to the palatal plane. They also reported an increase in transverse widths and mesiopalatal rotation of both right and left upper first molars.^3^

Grec et al.^4^, in their meta-analysis, compared the effects of intraoral distalizers with conventional and skeletal anchorage. They concluded that molar distalization was effective with both anchorage systems. The amount of distal molar movement was 3.34 mm with conventional anchorage and 5.10 mm with the skeletal anchorage system. The conventional anchorage system showed anchorage loss, represented by a premolar mesial movement of 4.01 mm^4^.

It is known that some intraoral palatal distalizers induce the rotation of upper first molars around the palatal root during distalization, due to a force passing palatally to the center of rotation of the molar^5^. Kinzinger et al.^6^ evaluated the biomechanics of a distal jet appliance, and reported that during the application of force palatal to the center of resistance (CR_es_) of the upper first molars, the teeth experienced therapeutically undesired mesial-inward and distal-outward rotation.^6^

To mitigate rotational movement and extend overall treatment duration, the approach of the present study utilizes a rigid and resistant distalizing mechanism, capable of counteracting molar rotation and intrusion, while also providing reliable anchorage post-distalization. Thus, the objective of this study was to assess the type of distal movement observed in upper teeth when utilizing a palatal distalizer with Variety SP^®^ screws anchored on palatal miniscrews.

## MATERIAL AND METHODS

Twenty patients, comprising three boys and fourteen girls aged between 12.5 and 24 years, were randomly chosen for bilateral upper molar distalization. The purpose of this intervention was to address dentoalveolar Class II malocclusion and dental arch length discrepancies.

The inclusion criteria encompassed patients with edge-to-edge Class II molar relationships and mild to moderate crowding in the maxilla. Exclusion criteria involved individuals with skeletal constriction of the maxilla, those who had undergone unilateral molar distalization, individuals with a history of prior orthodontic treatment, and those with insufficient oral hygiene.

All patients and their parents were provided with detailed information regarding the necessity of distalization and the associated procedures. Consent was obtained through the signing of an agreement form.

**Figure 1:**
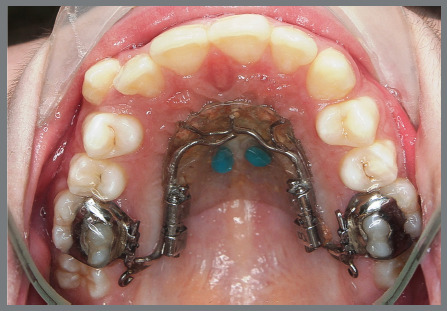
Bonded distalizer, with Variety SP^®^ screws.

**Figure 2:**
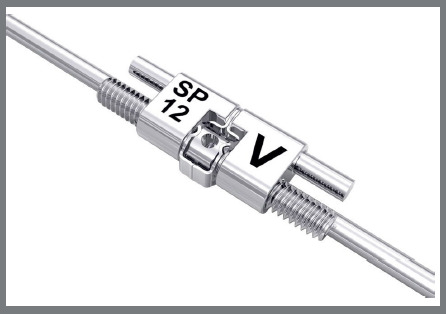
Variety SP^®^ screw, 12.0 mm in length.

The distalizing appliance comprised a palatal acrylic Nance button securely bonded to two palatal miniscrews. Utilizing two 12.0-mm long Variety SP^®^ expansion screws (Dentaurum GmbH & Co. KG, Turnstr. 31, 75228 Ispringen, Germany), positioned parallel to the alveolar crest and extending from the acrylic Nance button, the screws were welded to the upper first molar bands. The connecting wire had a diameter of 1.48 mm (Figs 1 and 2).

Preoperative mouth-rinsing with a 0.1% chlorhexidine solution was performed two days before and on the day of insertion. Local terminal anesthesia, using an adrenaline-free anesthetic, preceded the placement of the two miniscrews with neck and collar lengths of 8 mm and a diameter of 1.65 mm (Tomas, Dentaurum, Germany). The miniscrews were positioned in the paramedian region of the maxilla in the anterior palate area, using a manual screwdriver without predrilling.

Primary stability tests were conducted for all miniscrews. Three patients, experiencing palatal soft tissue inflammation after the initiation of distalization and requiring appliance removal, were excluded from the study. In four cases, upper third molars were extracted before the initiation of distalization. However, in the remaining thirteen cases, the upper third molars were in the germinating stage, eliminating the need for extraction.

Immediately after miniscrew insertion, impressions for the distalizing appliance confection were taken, using Zhermack Elite HD+ A-Silicone material. These impressions were promptly sent to a technician for the fabrication of the appliance.

It is commonly believed that titanium alloy miniscrews may undergo partial osseointegration approximately three weeks after insertion.^7^ ^,^ ^8^ Hence, our decision was to bond the appliance after this three-week period. Patients were instructed to activate the distalizing screws on each side with a quarter turn per week. In three cases, patients faced difficulties during self-activation, leading the orthodontist to perform the activations.

Lateral cephalograms were captured using Planmeca Promax 3D (Helsinki, Finland) before (T1) and after (T2) distalization for all ten patients.

**Figure 3:**
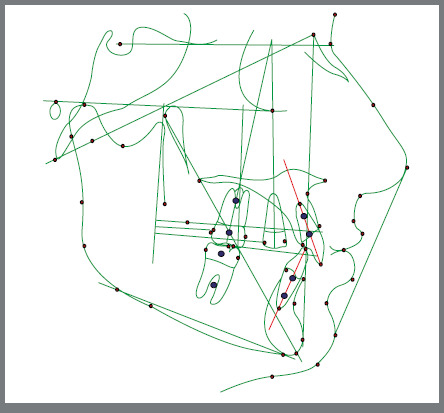
Lateral cephalogram tracing.

**Figure 4:**
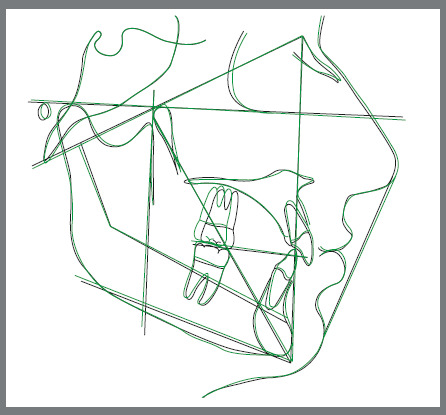
An example of lateral cephalogram tracings superimposition (T1 = black line, T2 = green line).

All lateral cephalograms were analyzed using Dolphin Imaging software by the same operator, who performed the analysis twice at a 2-week interval. In cases in which differences in measurements arose, the means of both readings were utilized in the subsequent statistical analysis (Figs 3 and 4).

The analysis involved calculating the potential movement of incisors and first premolars, as well as the relative distal movement of the first molars concerning the pterygoid vertical (PTV). Reference points for measurements were established as the most distal point of the crown for the upper first molars (U6) and first premolars (U4), along with the cement-enamel junction of the upper incisors (CEJ) (U1). To evaluate changes in the vertical plane of the upper first molars, the distance between the mesiobuccal cusp tip and the Frankfort horizontal plane was measured. In cases of overlapping structures, a midpoint between two points was marked for accurate assessment.

**Figure 5:**
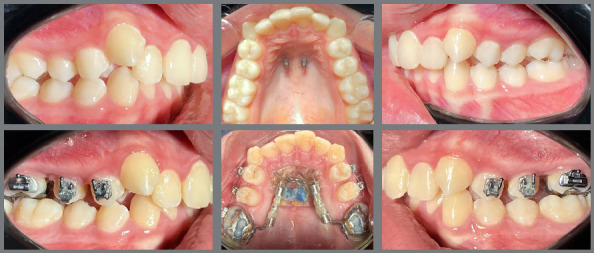
Intraoral photographs before (T1) and after distalization (T2).

Angular movements of U1, U4, and U6 were determined by measuring angles between the axis of teeth and the anterior cranial base. Distal tipping of the upper first molar was assessed as a decrease in the angle between the longitudinal axis of the molar and the cranial base. Conversely, for the upper first premolar and incisor, mesial tipping or flaring was reflected in the increase of the angle between the longitudinal axis of the teeth and the cranial base (Fig 5).

**Figure 6:**
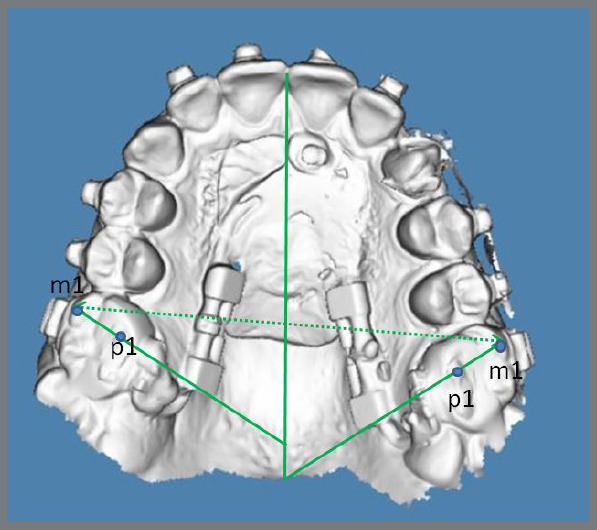
An example of cast analysis.

To assess upper first molar rotation and intermolar width, intraoral scans of the maxilla were taken for twelve patients at the start of treatment and immediately after the cessation of distalization. Intraoral scans were performed using the 3Shape TRIOS 3 intraoral scanner (3Shape A/S, Copenhagen, Denmark), and casts were generated with OrthoAnalyzer software (3Shape) (Fig 6).

A decrease in the angle between the line passing through the mesiobuccal cusp tip and mesiopalatal cusp tip and the palatal midline was indicative of first molar mesial-inward rotation. For intermolar width evaluation, the distance between the mesiobuccal cusp tips of the left (UL6) and right (UR6) first molars was measured, and an increase in this measurement indicated expansion. 

The calculated parameters are detailed in Table 1.

**Table 1:** Lateral cephalogram landmarks and measurements used in this study.

U4- dis. point /PTV: the distance between the most distal point of the maxillary first premolar to the pterygoid vertical
U6- dis. point /PTV: the distance between the most distal point of the maxillary first molar to the pterygoid vertical
U1 CEJ. /PTV: the distance between the cement-enamel junction of the maxillary central incisor to the pterygoid vertical
U1/SN: the angle between the axis of the maxillary central incisor and the anterior cranial base
U4/SN: the angle between the axis of the maxillary first premolar and the anterior cranial base
U6/SN: the angle between the axis of the maxillary first molar and the anterior cranial base
U6 mesial cusp/FH: The distance between mesial tip of the maxillary first molar and Frankfort horizontal

U4 = Upper first premolar, PTV = pterygoid vertical, U6 = Upper first molar, U1 = Upper incisor, CEJ = Cement-enamel junction, SN = Sella-Nasion line, mes.cusp = mesiobuccal cusp tip, FH = Frankfort Horizontal.

## STATISTICAL ANALYSIS

To assess the reliability of cephalometric measurements and cast analysis, two sets of measurements were taken for each participant, at two-week intervals. The possible error between the two sets of measurements was calculated using the Dahlberg formula.

Statistical calculations were conducted using SPSS software (version 14, SPSS, Chicago, Ill). The normality of the measured data was assessed using the Shapiro-Wilk test. Changes in each measurement from T1 to T2 underwent statistical analysis through a paired *t*-test analysis when a normal distribution was seen, while the Wilcoxon test was used for non-normally distributed data.

Differences with a significance level *p*< 0.05 (5%) were considered statistically significant. The relationship between distal tipping of upper molars and distal movement, as well as distal tipping and intrusion, was assessed using the Pearson correlation coefficient (r).

The primary objective was to reject the null hypothesis, thereby affirming the effective distalization of the upper first molars induced by the appliance.

## RESULTS

The mean distalization duration was 6.8 months. The possible error between the two sets of cephalometric measurements calculated using the Dahlberg formula, yielded a value of 1.1° for angular measurements and 0.8mm for linear measurements. For cast analysis, Dahlberg formula revealed possible error of 2.3° for angular and 0.6 mm for linear measurements. 

The results of T1 and T2 cephalogram analysis are shown in Table 2. Mean and standard deviations of T1 and T2 analysis are shown in Table 3.

**Table 2:** The results of T1 and T2 cephalogram analysis.

	U6/SN		U6-dis.point/PTV		U4/SN		U4-dis.point/PTV		U1 CEJ. /PTV		U1/SN		U6 mes. cusp/FH.	
	T1	T2	T1	T2	T1	T2	T1	T2	T1	T2	T1	T2	T1	T2
Patient 1	64°	63°	17.5 mm	12 mm	75°	61°	35 mm	33 mm	54.5 mm	56 mm	92°	89°	43.5 mm	43.5 mm
Patient 2	77°	74°	25 mm	22 mm	76°	75°	43 mm	44 mm	65 mm	65 mm	105°	98°	45 mm	45 mm
Patient 3	79°	74°	18.5 mm	15 mm	94°	89°	35 mm	36 mm	54 mm	54.5 mm	102°	104°	45 mm	44.5 mm
Patient 4	75°	60°	16.5 mm	12 mm	88°	79°	35 mm	34.5 mm	55.5 mm	56.5 mm	124°	125°	45 mm	42 mm
Patient 5	68°	64°	14.5 mm	12 mm	84°	87°	34 mm	34 mm	54 mm	53 mm	96°	92°	40 mm	39.5 mm
Patient 6	75°	59°	19.5 mm	17 mm	87°	85°	36 mm	35.5 mm	55.5 mm	56.5 mm	106°	102°	38.5 mm	37 mm
Patient 7	76°	72°	17 mm	15 mm	91°	88°	34 mm	33 mm	54 mm	54.5 mm	103°	105°	38 mm	38 mm
Patient 8	67°	52°	20.5 mm	16 mm	75°	72°	34 mm	33.5 mm	53 mm	53 mm	95°	91°	47 mm	44 mm
Patient 9	75°	76°	20.5 mm	14 mm	84°	83°	37 mm	37 mm	52 mm	52 mm	106°	102°	45.5 mm	46 mm
Patient 10	67°	54°	18 mm	13 mm	77°	64°	35 mm	33 mm	52 mm	53 mm	96°	97°	44.5 mm	42.5 mm
Patient 11	72°	73°	16.5 mm	13.5 mm	100°	90°	34 mm	32.5 mm	51 mm	50 mm	107°	104°	37 mm	37 mm
Patient 12	79°	76°	19.5 mm	16.5 mm	83°	78°	36 mm	35 mm	52.5 mm	53 mm	96°	98°	42.5 mm	41.5 mm
Patient 13	83°	81°	28 mm	24.5 mm	86°	83°	43 mm	42 mm	60 mm	60.5 mm	112°	115°	46 mm	45 mm
Patient 14	79°	70°	25 mm	22 mm	92°	85°	42 mm	42.5 mm	59.5 mm	60 mm	116°	112°	39.5 mm	38 mm
Patient 15	76°	73°	27 mm	23.5 mm	87°	83°	40.5 mm	39.5 mm	59 mm	58 mm	110°	106°	38 mm	37.5 mm
Patient 16	78°	73°	18.5 mm	14.8 mm	85°	70°	34 mm	33 mm	52.5 mm	52.5 mm	105°	101°	41 mm	41.5 mm
Patient 17	81°	75°	22.5 mm	17 mm	96°	82°	40 mm	38 mm	58 mm	58 mm	112°	106°	43.5 mm	42.5 mm

U4 = Upper first premolar, PTV = pterygoid vertical, U6 = Upper first molar, U1 = Upper incisor, CEJ = Cement-enamel junction, SN = Sella-Nasion line, mes. cusp = mesiobuccal cusp tip, FH = Frankfort Horizontal, T1 = before distalization, T2 = after distalization.

**Table 3:** T1 and T2 cephalogram and cast data statistical analysis.

Cephalometric and cast analysis	n	T1 mean	T1 SD	T2 mean	T2 SD	T1-T2 mean	T1-T2 SD	p-value
U6/SN (degrees)	17	74.7	5.4	68.7	8.4	6.0	3.0	0.0004
U6-dis.point./PTV (mm)	17	20.2	3.9	16.4	4.1	3.8	0.2	< 0.0001
U4/SN (degrees)	17	85.9	7.36	79.6	8.6	6.2	1.24	0.0002
U4-dis.point./PTV (mm)	17	36.9	3.36	36.2	3.7	0.68	0.34	0.0098
U1 CEJ. /PTV (mm)	17	55.41	3.8	55.64	3.9	-0.23	0.1	0.14
U1/SN (degrees)	17	104.8	8.4	102.2	9.5	2.65	1.1	0.028
U6 mes. cusp/FH (mm)	17	42.3	3.2	41.4	3.0	0.88	0.2	0.006
UR6 m1-UL6 m1 (mm)	12	50.87	3.56	53.4	3.57	2.54	0.01	< 0.0001
UR6 m1p1/midline (degrees)	12	55.7	10.14	51.6	9.95	4.1	0.19	0.017
UL6 m1p1/midline (degrees)	12	55.5	7.4	52.1	7.5	3.4	0.1	0.021

U4 = Upper first premolar, PTV = pterygoid vertical, U6 = Upper first molar, U1 = Upper incisor, CEJ = Cement-enamel junction, SN = Sella-Nasion line, T1 = Before distalization, T2 = After distalization, UR6 = Upper right first molar, UL6 = Upper left first molar, m1 = Mesiobuccal cusp tip, p1 = Mesiopalatal cusp tip.

Significant differences in distal movement and distal tipping of the upper first molars were found. According to measurements, the upper first molars’ mean distal movement (U6-dis.point/PTV) and distal tipping (U6/SN) were 3.8 mm (*p*<* *0.05) and 6.0° (*p*<* *0.05), respectively. In twelve patients, the mean distal tipping of the upper molars was 2.8* *±* *0.45° (*p*<* *0.05) and in five cases, the molars were tipped distally at a mean of 13.6* *±* *1.63° (*p*<* *0.05). In two of twelve patients, a small root movement of the upper first molars was found. 

The upper first premolars tipped distally (U4/SN) on average 6.2 ± 1.24° (*p*< 0.05), and the mean change in distance between U4-dis.point and PTV was 0.68 ± 0.34 mm (*p*< 0.05). The distance from the cement-enamel junction of the maxillary central incisor to the PTV point (U1 CEJ./PTV) was increased in some patients and decreased in others, but the mean change was -0.23 ± 0.1 mm (*p*> 0.05). On the other hand, the maxillary central incisors were tipped palatally 2.65 ± 1.1° (*p*< 0.05).The mean change in distance between the mesiobuccal cusp tip of the maxillary first molar and the FH was 0.88 ± 0.2 mm (*p*< 0.05). In twelve cases, the distance remained unchanged or exhibited statistically insignificant differences, with a mean 0.29 ± 0.04 mm (*p*> 0.05). However, in five cases, intrusion of the upper first molars was observed, with a mean of 2.3 ± 0.79 mm (*p*< 0.05). 

The Pearson correlation coefficient (r) between upper first molar distal tipping and intrusion was 0.47. Additionally, the Pearson correlation coefficient (r) between upper first molar distalization and distal tipping was -0.0052.

Distance between mesiobuccal cusp tips of upper right and left first molar increased a mean of 2.54 mm (*p*< 0.05). The angle between mesiobuccal cusp-mesiopalatal cusp tips and palatal midline decreased a mean of 4.1 ± 0.19° (*p*< 0.05) and 3.4 ± 0.1° (*p*< 0.05) for UR6 and UL6 respectively.

## DISCUSSION

In twelve cases, the upper first molars moved distally almost bodily, with minor distal tipping. However, in five cases, the molars tipped distally significantly, in conjunction with distal movement. The biomechanical explanation could be the force passing occlusal to the CR_es_ of the upper molars. According to Gandhi et al.^9^, the CR_es_ of the maxillary first molar is situated apically and distally to the trifurcation area. Its specific location can vary among different patients. To ensure controllable movement, it is crucial to consider the tooth CR_es_ during the process of distalization. However in some cases, due to insufficient palatal depth, technicians find it challenging to place distalizing screws deep enough for the force to pass near the CR_es_ of the first molars.

In the present study, we investigated the relationship between upper first molar distal tipping and distal movement. The Pearson correlation coefficient (r = -0.0052) suggests a very week linear relationship between upper first molar distal tipping and distal movement. This suggests that the degree of distal tipping was not significantly associated with the amount of distal movement in the present study sample.

Distal tipping of the upper first molar is more pronounced when the second molar is in the germinating phase.^10^ In the present study, ten cases involved fully erupted second molars, while in seven cases, the second molars were in the eruption phase. In these seven cases, we observed distal movement with small distal tipping of the upper first molars.

Kircali et al.^11^ evaluated the dentoalveolar and dentofacial effects of a miniscrew-supported pendulum appliance during upper molar distalization, reporting 4.2 mm of first molar distalization, significant distal tipping of 8.9°, and a significant intrusion of 0.6 mm. In another study, Cozzani et al.^12^ reported 4.7 ± 1.6 mm of distalization, 2.8° of distal tipping, and 0.7 mm of intrusion in the upper first molars using a group of miniscrew-supported distal jet appliances. Chiu et al.^13^, in their comparison of two intraoral molar distalization appliances (distal jet versus pendulum), reported 1.7 ± 1.4 mm of extrusion of the U6 in the distal jet group and 1.6 ± 1.2 mm in the pendulum group. In another study, Tekale et al.^14^ reported 0.25 mm of intrusion movement on the distal cusp of the maxillary first molar and 0.14 mm of extrusion movement on the mesial cusp of the first molar in the Z direction. Kinzinger et al.^3^ reported a mean extrusion of the first molar of 0.63 ± 0.70 mm. In another study, Kinzinger et al.^6^ evaluated the biomechanics of a distal jet appliance, indicating that a -21 cN intrusive force acted on the upper first molar during distalization. This force remained stable until 1-mm of distal movement, after which it consistently dropped.

In our study, when the force passed near the CR_es_ of the first molar, we observed vertical movement that was statistically insignificant. However, in five cases, when the force passed occlusal to the CR_es_, we observed significant intrusion along with distal tipping. The occurrence of intrusion during distal tipping of the molar suggests a complex biomechanical interplay. To gain a deeper understanding of this phenomenon, further investigation through finite element analysis is recomended.

In a systematic review, Fiorillo et al.^15^ assessed eleven studies and concluded that upper molars can be effectively distalized with Temporary Anchorage Device (TAD)-assisted distalizing appliances, achieving a range of 3.0 to 5.3 mm. Spontaneous distal migration of upper premolars was observed, ranging from 1.65 to 4.30 mm. In our study, distal migration was 0.57mm, but the distal tipping was 5.13°. The only plausible explanation for distal tipping with minor migration is the growth-induced changes observed in the majority of patients included in this study.^16^ The distal tipping of upper premolars can be attributed to the use of a direct skeletal anchorage system in our study, where premolars were not included in the anchorage unit. They tipped distally due to the stretching of transseptal fibers.^17^

To prevent anchorage loss, the appliance in this study was supported by two palatal miniscrews. Grec et al.^4^ concluded in their study that intraoral distalizers with skeletal anchorage, unlike traditional systems, were more effective for molar distalization and showed no anchorage loss. Moreover, the use of direct skeletal anchorage induced spontaneous distal movement of upper premolars. Ludwig et al.^18^ suggested that the anterior palate is a reliable zone for miniscrew insertion. In our study, miniscrews were consistently placed 3-4 mm parasagittal to the midpalatal suture in all patients.

In nine cases, the distance U1 CEJ./PTV was increased; and in six cases, U1/SN angle was increased. Given the use of direct skeletal anchorage, the only plausible explanation for the increase in results could be the growth-induced changes and possible errors during measurements.^16^

Another possible reason could be the fact that in several cases brackets were bonded to upper teeth before the completion of distalization.

Another important factor that needs consideration during distalization is the stiffness of the distalizing appliance.

In their systematic review, Ceratti et al.^19^ compared rigid and non-rigid distalizers, and concluded that non-rigid appliances tipped upper molars more than rigid appliances, although the amounts of molar distalization and intrusion were similar. In our study, we utilized 12-mm expansion screws (Dentaurum, Variety SP^®^). According to the manufacturer, the expansion per turn is 0.8 mm. Lombardo et al.^20^, comparing different palatal expanders, reported that the Dentaurum Variety SP^®^ screw body size was 9.6 × 5 × 3mm, with a maximum expansion of 12 mm, and an arm diameter of 1.48 mm. From the initial activations, the screw exerted a force of 302 N (Newtons), and after 10 and 15 activations, it remained over 250 N. The stiffness of the screw remained high after 6-8 activations. This could be another explanation for the very small amount of distal tipping observed in five patients. Moreover, the stiffness of the “system” allowed us to use Class III elastics (as anchorage for lower molar uprighting mechanics, when necessary) or other elastic module from upper first molars, for the retraction of upper premolars and canines without fear of mesial tilting of upper molars.

In a study by Cozzani et al.^12^, the mean distalization time was 9.1 ± 2.8 months. Kinzinger et al.^3^ reported a treatment duration of 6.7 months. The average distalization time with the Dual-force distalizer (DFD) was 5 months.^21^ In our study, the mean distalization time was 6.8 months.

Kinzinger et al.^3^ reported mean increase in transverse width of 1.79 ± 1.08 mm between the mesiobuccal cusps, 2.58 ± 0.69 mm between the central fossae, and 3.03 ± 0.68 mm between the distobuccal cusps. Additionally, the upper first molar rotated mesiopalatally a mean of 8.35 ± 7.66° on the right side and 7.88 ± 5.50° on the left side. 

Kircali et al.^22^ reported an increase in the distance between mesiobuccal cusps of the upper first molars by 2.4 mm, along with insignificant mesiopalatal rotations of the upper first molars. 

Kinzinger et al. ^6^, when evaluating the biomechanics of the distal jet appliance, stated that, due to the force passing palatal to the CR_es_ of the upper first molars, they experienced a mesially rotating moment, that decreased during the course of distalization (700 cN/mm initially; 200 cN mm after 3 mm).

Kang et al.^23^ compared the results of distalization of the first molars with a modified palatal anchorage plate (MPAP) and a miniscrew-anchored pendulum appliance. They stated that in both methods, distalization was accompanied by intrusion, buccal tipping, and mesial-in rotation.

Increase in intermolar width in our study was similar to the results of above-mentioned studies, but despite our efforts to mitigate rotational effects during molar distalization, by employing a distalizing mechanism designed to be rigid and resistant, we observed a mean rotation of 4.1°(*p*<0.05) for the upper right molars and 3.4°(*p*<0.05) for the upper left molars. These findings indicate that, while our approach aimed to provide stability and prevent undesired molar rotation, some degree of rotational movement still occurred during the treatment, due to palatally acting force. Further investigation is recommended to better understand the factors contributing to this rotational effect and to refine treatment protocols accordingly.

## CONCLUSION

This study demonstrates the effectiveness of the palatal distalizer with Variety SP^®^ screws for bilateral molar distalization without anchorage loss. Careful planning of the line of the acting force during appliance fabrication is crucial.

» When the force passes near the center of resistance (CR_es_) of the upper first molars, the orthodontist can expect distal movement with minor distal tipping in the sagittal plane, due to the stiffness of the system, even if second molars are in the germinating stage and can act as a fulcrum.
» When the force is applied near the CR_es_ of the upper first molar, minimal vertical changes occur.
» When the force passes occlusally beyond the CR_es_, it induces significant distal tipping and intrusion.
» Due to the force acting palatally to the center of resistance of the upper first molars, some degree of mesial-inward rotation of the molars took place.

The results are based on records of only seventeen patients, needing further investigation with a larger sample size. Future research should specifically address upper first molar torque changes, second molar movement, the amount of acting force, and the effect of upper third molars on distalization.
